# Henoch–Schönlein Purpura Associated with Lung Cancer: When Paraneoplastic Manifestations Impede Oncological Management

**DOI:** 10.1155/2021/8847017

**Published:** 2021-02-06

**Authors:** Éloïse Philippe, Aude Barnier, Juliette Menguy, Gilles Robinet, Gilles Quéré, Francis Couturaud, Renaud Descourt

**Affiliations:** ^1^Institut De Cancérologie, CHU Morvan, Brest 29200, France; ^2^Service De Pneumologie, EA3878-GETBO, Université De Bretagne Occidentale, CHU Cavale Blanche, Boulevard Tanguy-Prigent, Brest 29200, France

## Abstract

**Background:**

Henoch–Schönlein purpura (HSP) is an uncommon syndrome that mostly occurs in children, in whom it is frequently triggered by infections. In contrast, HSP in adults is more frequently of neoplastic origin. *Case Presentation*. We report HSP associated with a locally advanced lung squamous cell carcinoma that was considered a paraneoplastic syndrome. Systemic corticosteroids were given because a kidney biopsy revealed active glomerulonephritis. Concomitant chemoradiotherapy achieved a partial response of the lung tumor. Consolidation immunotherapy (programmed death protein-1-ligand-1 (PD-L1) inhibitor) was cancelled because HSP is known to be an autoimmune vasculitis, and long-term corticosteroid therapy was pursued.

**Conclusion:**

Further prospective studies are needed to evaluate the effect of anti-PD-(L) 1 immunotherapies on autoimmune manifestations.

## 1. Introduction

Henoch–Schönlein purpura (HSP) is an uncommon syndrome rarely observed in adults. Its association with cancer raises the question of a causal link. Its clinical manifestations most frequently involve skin, joints, gastrointestinal tract, and glomeruli. We describe this paraneoplastic vasculitis associated with lung squamous cell carcinoma (SCC).

## 2. Case Presentation

**Figure 1:**
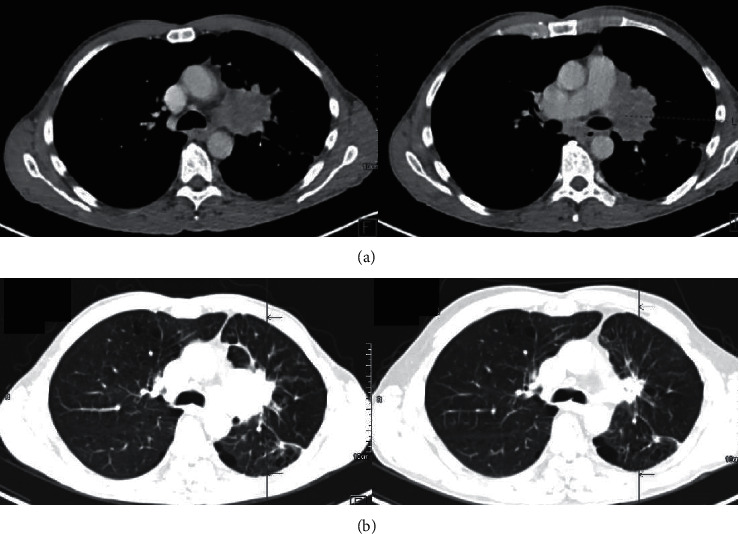
CT scans showing (a) left upper lobe SCC at diagnosis and (b) a partial response to chemoradiotherapy at 4 weeks.

**Figure 2:**
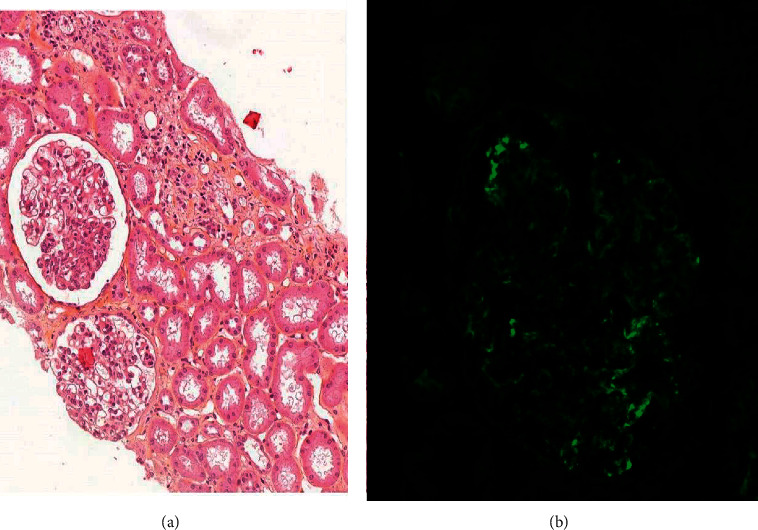
Histology of a kidney biopsy. (a) Endocapillary proliferation (stained with hematoxylin-eosin-saffron; ×300). (b) Immunofluorescent labeling of IgA deposits showing a diffuse, granular, parietal pattern (×400).

A 59-year-old man, an active smoker with chronic bronchitis, consulted for a cough that had persisted for several weeks. His chest computed tomography (CT) scan revealed a spiculated mass in the left upper lobe infiltrating the mediastinum (Figure 1(a)). Histology of bronchial biopsies revealed SCC. Ten days later, he was hospitalized for a fever that persisted despite first-line antibiotics, associated with an inflammatory syndrome (C-reactive protein, 51 mg/L; leukocytes, 11.5 G/L). Vascular purpura of the lower extremities appeared on hospitalization day 2. The skin biopsy showed leukocytoclastic vasculitis with immunoglobulin A deposits. Hyperthermia worsened and he experienced an impure nephrotic syndrome (acute renal failure, hematuria) on day 5 (proteinuria 5 g/24 h). The kidney biopsy showed proliferative glomerulonephritis with IgA deposits, suggesting renal localization of HSP (Figure 2). Other vasculitides were excluded because all immune markers tested were negative (Table 1). Systemic corticosteroids were started to treat active histological lesions (500 mg/d solumedrol bolus for 3 days, followed by progressive tapering over a total duration of 6 months). Proteinuria rapidly decreased to 1.53 G/24 h over 10 days. Fever and skin rash resolved in 7 days. Based on the ^18^F-fluorodeoxyglucose-positron-emission tomography (FDG-PET) scan, the tumor was ranked cT4N2M0. Concomitant chemoradiotherapy (CRT) (3 cycles of cisplatin-vinorelbine and 66 Gy of radiation (2 Gy/fraction) over a total treatment duration of 8 weeks) achieved a partial response at 4 weeks (Figure 1(b)). Consolidation immunotherapy with antiprogrammed death protein-1-ligand-1 (PD-L1) (durvalumab) after CRT was discussed in a multidisciplinary meeting but excluded because of the initial autoimmune manifestations and the ongoing corticosteroid therapy. Twelve months after completing CRT, a follow-up CT scan showed enlargement of the irradiated residual tumor. Histology of fibroscopy-obtained bronchial biopsies found SCC infiltration of a left superior lobar exophytic bud and FDG-PET scan indicated hypermetabolism of the lung mass with no sign of distant metastases, thereby confirming localized recurrence. The latter was not associated with a vasculitis relapse.

We obtained the patient's written informed consent for publication.

## 3. Discussion

HSP or rheumatoid purpura is a systemic small-vessel vasculitis associated with tissue deposits of IgA immune complexes. An external antigenic attack inducing an abnormal response of a faulty or immature immune system might be the pathophysiological mechanism. Several organs may be affected, with symptomatic deposits in skin, joints, and gastrointestinal tract being the most common.

The outcome is mainly conditioned by renal involvement. HSP is considered to be a childhood pathology but can occur in adults, sometimes associated with solid-organ malignancies [1]. Abnormal deposits might be secondary to an immune reaction against tumor antigens. Failure to clear immune complexes and similarity of tumor antigens and endothelial cell antigens have also been advanced. Although the cancer-HSP relationship is still unclear, several case reports have described this association.

About 5% of vasculitis patients have associated cancer (2/3 hematological malignancies and 1/3 solid cancer). Vasculitis regression during cancer treatment is commonly considered evidence of a paraneoplastic syndrome. Paraneoplastic vasculitides are uncommon (frequency 1/80,800) [2], among which only 6.8% are HSP. When paraneoplastic vasculitides occur, they are often associated with lung cancer, especially SCC or small cell lung cancer [3]. A recent systematic review reported 10 patients with both lung cancer (two small-cell lung cancers, three adenocarcinomas, four SCCs, and one with unspecified non-small-cell lung cancer) and cutaneous vasculitis [4]. Paraneoplastic purpura in our patient is plausible. An infectious origin was excluded based on clinical improvement with corticosteroids alone. Other possible triggering factors were also excluded (medication, allergic reaction, and another autoimmune disease) (Table 1).

Autoimmune paraneoplastic manifestations raise doubt as to the indication of immunotherapy for advanced or locally advanced non-small-cell lung cancer. The beneficial impacts of immunotherapy on overall survival and progression-free survival are known [5–7], but patients with preexisting autoimmune disease were excluded from clinical trials and early access programs. Retrospective series suggests that worsening of preexisting autoimmune conditions is common in lung cancer patients treated with anti-PD-(L) 1 and that immunosuppressants (like corticosteroids) may lower the efficacy of immunotherapies [8]. Current data are controversial regarding the use of immune-checkpoint inhibitors in patients with preexisting autoimmune disease. A recent nationwide, multicenter, cohort study assessed the safety and efficacy of immune-checkpoint inhibitors as cancer therapy for patients with preexisting autoimmune diseases [9]. According to its results, the occurrence of an immune-related adverse effect and/or flare of preexisting autoimmune disease was frequent (71%) but mostly manageable without immune-checkpoint inhibitor discontinuation.

In conclusion, as the indications of immunotherapy become more and more numerous, further studies are needed to evaluate the efficacy and overall safety of immunotherapy for patients with advanced or locally advanced non-small-cell lung carcinoma and preexisting or concomitant paraneoplastic autoimmune disease.

## Figures and Tables

**Table 1:** Serology results for autoimmunity.

Autoimmune-testing target	Result
Antinuclear antibodies	Positive (1/320)
Anti-DNA antibodies	Negative
Histone antibodies	Negative
Anticentromeres antibodies	Negative
Antibasement membrane antibodies	Negative
Rheumatoid factor	Negative
Polynuclear anticytoplasmic antibodies	Negative
Serum free light-chain assay	Normal
Complement protein assay	Normal

## Data Availability

The clinical data used to support the findings of this study are included within the report.

## Abbreviations

HSP: Henoch–Schönlein purpura
CRT: Chemoradiotherapy
CT: Computed tomography
FDG-PET: ^18^F-fluorodeoxyglucose-positron-emission tomography
PD-L1: Antiprogrammed death protein-1-ligand-1
SCC: Squamous cell carcinoma.
